# VE-cadherin shedding in vitro and in patients with aortic aneurysm and dissection

**DOI:** 10.1038/s41598-024-77940-3

**Published:** 2024-11-05

**Authors:** Paul Stammer, Inka Terhorst, Jiangang Guo, Abdulhakim Ibrahim, Alexander Oberhuber, Thorsten Eierhoff

**Affiliations:** 1 Clinic for Vascular and Endovascular Surgery, University Hospital Münster, Albert-Schweitzer-Campus 1, 48149 Münster, Germany; 2 Present Address: Department of Endovascular and Vascular Surgery, Affiliated Hospital of Guilin Medical University, Guilin, Guangxi China

**Keywords:** VE-cadherin, Aortic disease, ADAM10, TNF-α, Microbiota, Aortic diseases, Cell biology, Molecular medicine

## Abstract

VE-cadherin (VEC) is a major endothelial adhesion protein, which controls vascular homeostasis. During vascular diseases, VEC can be shed from the endothelial surface by proteases like ADAM10/17, which cleave the extracellular domain of VEC in response to inflammatory cytokines like TNF-α. The resulting, soluble fragments (sVEC) are discussed as a potential marker for endothelial barrier breakdown. However, its pathologic role or its potential as a specific biomarker for aortic diseases is yet unknown. Here we investigated the specificity and linkage of sVEC production with ADAM10/17 and TNF-α, both in vitro and in patients with aortic aneurysms and dissections, comparing the findings with those from patients with carotid stenosis and varicosis. Thereby, the baseline levels of sVEC, TNF-α, ADAM10 and Albumin was measured in clinical plasma samples and cell culture supernatants of human aortic endothelial cells (HAOEC) treated with TNF-α or ADAM10/17 inhibitors. The integrity of HAOEC monolayers was tested by permeability assays using Alexa488-conjugated dextran (10 kDa). Peripheral EDTA plasma samples taken preoperatively from patients ≥ 18 years of age that were diagnosed for aortic dissection (*n* = 29), aortic aneurysm (*n* = 76), carotid stenosis (*n* = 29) and varicose veins (*n* = 24) were included. In vitro shedding of VEC was induced by TNF-α and depends on ADAM10/17, which led to altered endothelial permeability. Absolute plasma sVEC levels in patients with aortic dissection (3016 ± 1008 ng/mL) and aneurysm (3288 ± 1376 ng/mL) were not statistically significantly different from patients with carotid stenosis (3013 ± 687.6 ng/mL) and varicose veins (3313 ± 1337 ng/mL). Plasma sVEC levels correlated positively with plasma TNF-α (*r* = 0.5586, *p* < 0.0001) and ADAM10 (*r* = 0.7003, *p* < 0.0001) levels with the highest degree of correlation between ADAM10 and sVEC for chronic aortic dissection (*r* = 0.7890, *p* = 0.0013), reflecting TNF-α and ADAM10 dependency of VEC shedding. In summary, VEC shedding and (plasma) sVEC levels are influenced by TNF-α and ADAM10/17 and could play a relevant role in the specific pathophysiological context of aortic diseases.

**Supplementary Information:**

The online version contains supplementary material available at 10.1038/s41598-024-77940-3.

## Introduction

The integrity of the vascular endothelium is of particular relevance. Endothelial dysfunction, for example, because of increased endothelial permeability (EP) often occurs in the early stages of many arterial diseases, manifesting as aneurysms and dissections, among others^1^.

VE-cadherin (VEC), an approximately 130 kDa transmembrane protein, is involved as a signaling and adhesion molecule in the establishment of cell-cell contacts and thus in the regulation of vascular permeability. For this purpose, the extracellular domains of VEC interact, in a Ca^2+^-dependent manner, with the opposing extracellular VEC domains of neighboring cells, forming adherence junctions (AJs)^2^. In vascular endothelial cells, VEGF, inflammatory molecules and cytokines such as LPS and TNF-α, induce phosphorylation of VEC and activation of matrix metalloproteases (MMP) ADAM10, ADAM17, MMP2 and MMP9. This leads to the endocytosis of VEC, proteolysis of its cytoplasmic domain and shedding of its soluble ectodomain (sVEC), ultimately increasing in EP^3,4^. Notably, sVEC itself can inhibit angiogenesis and cause endothelial barrier dysfunction^5,6^.

Interestingly, metalloproteases involved in the processing and shedding of VEC are highly expressed in ruptured atherosclerotic lesions and aortic aneurysms^7^. Recently, it was found that activation of ADAM10 promotes a coagulation factor XIa (FXIa)-dependent shedding of VEC, which may have implications for patients with inflammatory diseases^8^. This raises the question of whether ADAM10 cleavage products, particularly of VEC, can be detected as an indicator of endothelial damage in the plasma of the respective patients. This could provide valuable information about the condition and potentially the course of the disease. Indeed, it is currently discussed, although in a different context, whether sVEC may act as a marker of endothelial barrier damage in patients with critical organ dysfunction. Thus, increased plasma levels of sVEC have been demonstrated in patients with systemic inflammation and sepsis, particularly in patients with acute renal injury^9^.

CT-based visualization is still a gold standard for diagnosis and control of aortic disease^10^, though circulating biomarkers may be more specific and sensitive towards the onset and progression of the disease. Although general biomarkers, like D-dimer are known^11^, specific ones are still lacking for clinical applications. In the context of acute or chronic aortic pathologies, sVEC as a marker is poorly investigated. So, qualifying data to assess the potential of sVEC for the detection of aortic pathologies and discrimination from other vascular diseases or even for its translation into IoT applications for real-time healthcare monitoring^12^ are missing. Recently Wang et al. reported elevated serum levels of sVEC and vinculin and discussed these as markers for acute type B aortic dissections^13^. Other studies show that ADAM17 is involved in the progression of human thoracic aortic aneurysm (TAA) and is likely associated with increased EP through the shedding of VEC in vitro^14^. The involvement of ADAM17 and other matrix metalloproteases in acute and chronic aortic diseases^14–16^, with VEC among their substrate targets, suggests that sVEC is formed at the aortic wall and subsequently released into the bloodstream. Thus, sVEC could act as a marker of endothelial injury in aortic disease that correlates with disease severity and progression. Moreover, research exploring factors influencing sVEC shedding could contribute to a better understanding of the pathophysiological role of VEC proteolysis and its regulation in the context of acute and chronic aortic diseases.

Here we investigate the influence of TNF-α and ADAM 10/17 on the shedding of VEC in HAOEC which is supplemented by an analysis of clinical plasma samples of patients with different vascular diseases, including Stanford type B aortic dissection and aneurysm to evaluate if baseline sVEC levels are specific to aortic pathologies.

## Results

### TNF-α and ADAM10/17 impact VEC shedding and endothelial permeability of HAOEC

**Fig. 1:**
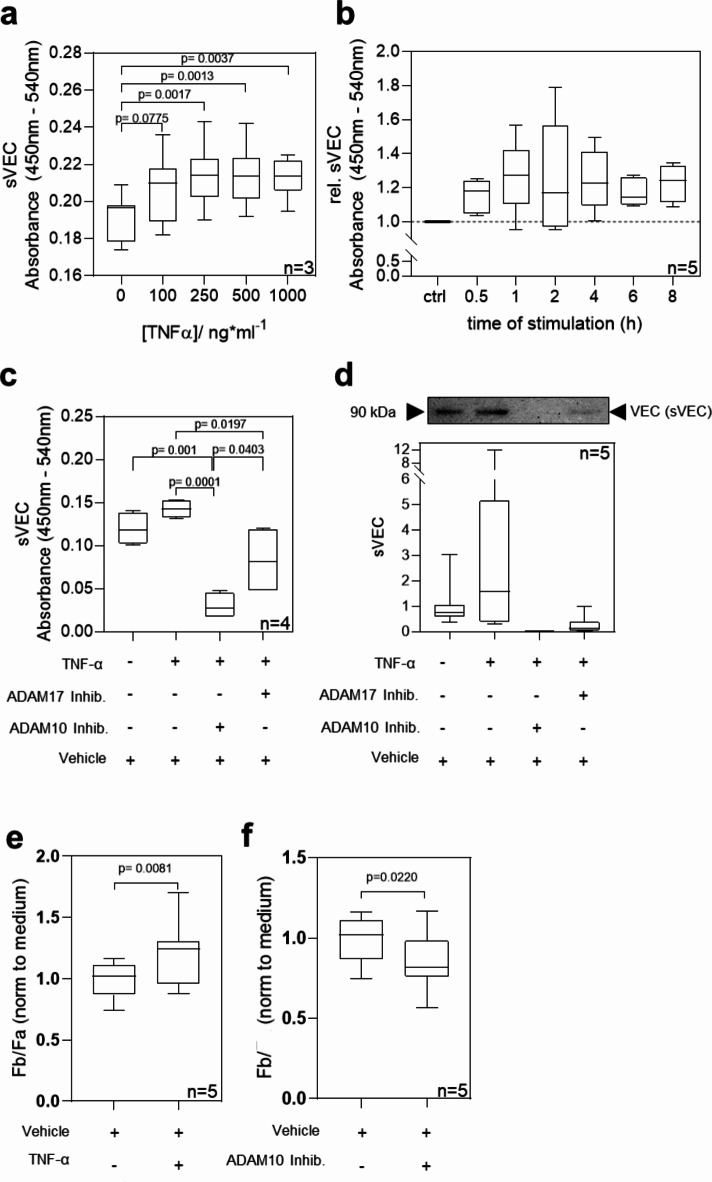
TNF-α and ADAM10/17 trigger shedding of VE-cadherin (VEC) in human, aortic endothelial cells (HAOEC). (**a**) soluble VEC (sVEC) levels detected by ELISA in culture supernatants of HAOEC stimulated with various concentrations (for 4 h). (**b**) Relative sVEC levels in the culture supernatant of HAOEC treated with TNF-α (100 ng/mL) for the indicated times. Absorbance was normalized to the absorbance measured in untreated (ctrl) cells (dashed line) **c**,** d**) sVEC levels read out by ELISA (**c**) or Western Blotting (**d**) against VEC in supernatants of HAOEC stimulated with TNF-α (100 ng/mL, 2 h) in the presence or absence of pharmacological inhibitors for ADAM10 and ADAM17 (each 10 µM). **d** (lower panel) densitometric quantification of 90 kDa fragment of VEC. The uncropped blot is shown in the Supplementary Fig. 4a. **e**, **f** Diffusion of Alexa488-conjugated Dextran (10 kDa) through a confluent monolayer of HAOEC treated for 4 h with TNF-a (**e**) or ADAM10 inhibitor (**f**). A minimum of *N* = 3 independent experiments were conducted. P values were determined using one-way analysis of variance (*ANOVA*) (**a-d**) and unpaired t-Test (*Welch’s t-test*) for **e**,** f**.

Treatment of HAOEC with various concentrations of TNF-α between 0 and 1000 ng/ml considerably increased sVEC specific absorbance in the cell-cleared, culture supernatant by ≥ 1.08-fold of control for TNF-α ≥ 100 ng/mL, reaching a plateau for A_450nm-540 nm_ of 0.21 (Fig. 1a). Thereby, the tested concentrations of TNF-α cause no impairment of cell viability as measured by the conversion of MTT tetrazolium into formazan (Supplementary Fig. 1). When testing different incubation times between 0.5 and 8 h with 100 ng/ml TNF-α, mean sVEC absorption was elevated by about 1.2-fold of untreated cells without significant fluctuations during the tested period (Fig. 1b).

## Inhibition of ADAM10 and ADAM17 attenuates influence of TNF-α on VEC shedding

When TNF-α was co-incubated with specific inhibitors against ADAM10 and ADAM17, sVEC was no longer elevated in the cell culture supernatant. Instead, we observed a significant decrease in sVEC absorbance by 4.7-fold when applying the ADAM10 inhibitor compared to HAOEC treated with TNF-α alone (Fig. 1c). Moreover, under influence of ADAM10/17 inhibitors, resulting sVEC levels were even lower than observed for the basal condition. Of note, application of the ADAM17 inhibitor resulted in a less pronounced, but still considerable attenuation of TNF-α triggered VEC shedding as indicated by a 1.7-fold lower mean absorbance of sVEC (Fig. 1c).

Complementary to ELISA we probed culture supernatants of HAOEC by Western Blotting using an antibody against the extracellular domain of VEC. Thereby we confirmed the presence of a VEC band with an apparent molecular weight of 90 kDa, corresponding to the shed extracellular domain of VEC, whereas full-length VEC (approx. 130 kDa) could not be detected in the supernatants (Fig. 1d, upper panel). Densitometric quantification of the corresponding sVEC bands confirmed a significantly attenuated TNF-α-induced VEC shedding upon inhibition of ADAM10/17 that was slightly more pronounced upon ADAM10 inhibition (Fig. 1d, lower panel).

As shedding of VEC may interfere with junctional integrity, we tested if TNF-α and inhibition of ADAM10 influence the integrity of a HAOEC monolayer. Thereby we analyzed the permeation of Alexa488-Dextran (10 kDa) through the cell monolayer upon stimulation with TNF-α (100 ng/mL) or pharmacological inhibition of ADAM10. We found that TNF-α stimulation of HAOEC for 4 h significantly increased the mean permeability of the monolayer for Alexa488-Dextran by a factor of 1.2 (Fig. 1e), whereas inhibition of ADAM10 decreased endothelial permeability compared to a vehicle-treated endothelial monolayer (Fig. 1f).

Our findings suggest that TNF-α and ADAM10/17 mediate shedding of VEC which correlates with altered endothelial barrier function. Since both TNF-α and ADAM10 are involved in aortic pathologies, they may be associated with VEC shedding in the context of aortic pathologies. Therefore, we next tested plasma samples of patients with aortic aneurysms or Stanford type B aortic dissections for sVEC levels.

## Quantification of plasma sVEC levels in patients with vascular diseases

**Fig. 2:**
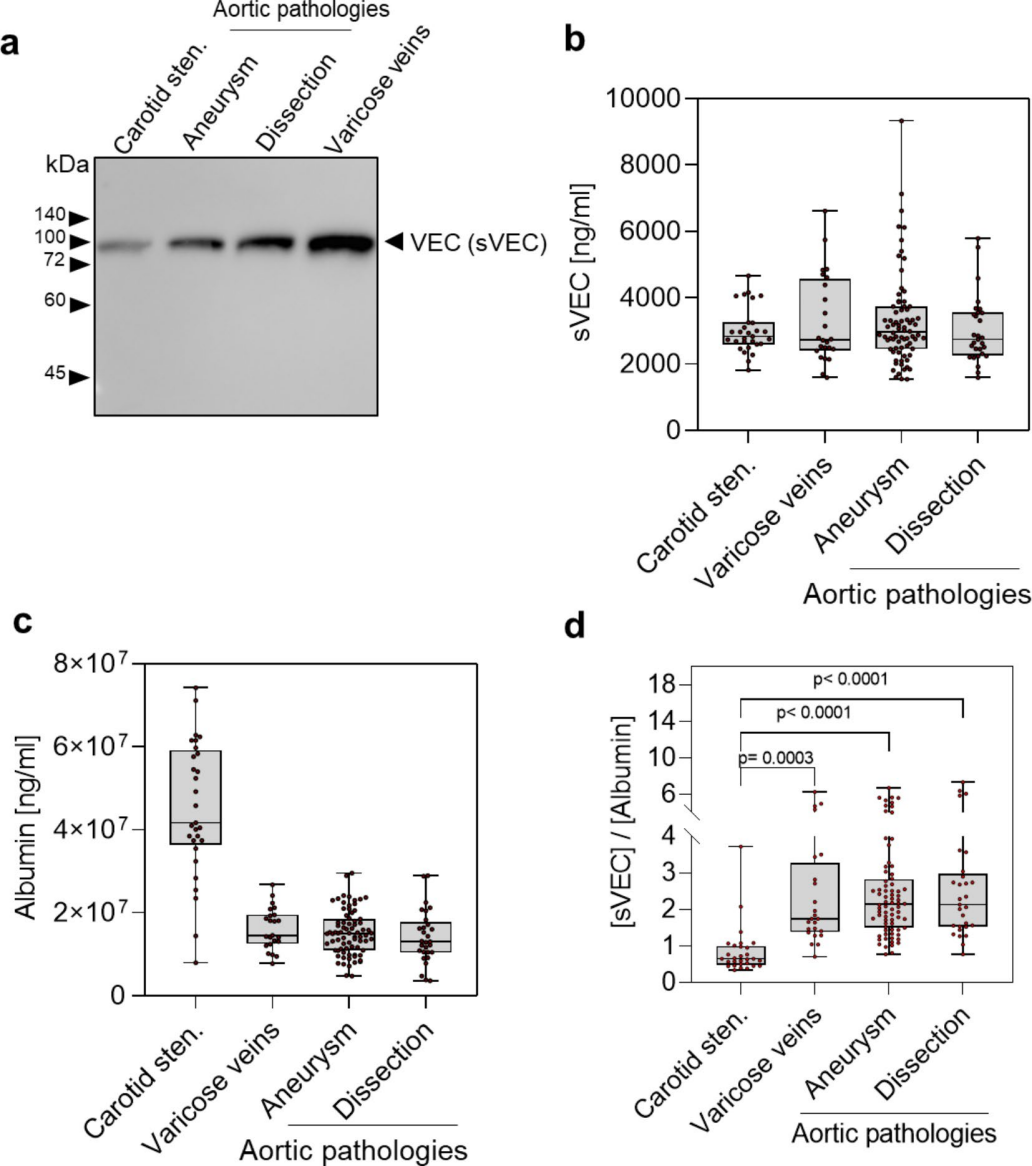
Plasma sVEC levels in patients with different vascular diseases. (**a**) Western Blot analysis of plasma samples of patients with indicated disease using specific antibody against VEC. The uncropped blot is shown in the Supplementary Fig. 4b. (**b**,** c**) Absolute plasma sVEC and albumin levels determined by ELISA for given vascular diseases. (**d**) Albumin-normalized sVEC levels calculated as (sVEC [ng/ml]) / (albumin [ng/ml]) * 10000. A total of *N* = 158 plasma samples of individual patients was analyzed (Carotid stenosis: *N* = 29; Varicose veins: *N* = 24; Aortic aneurysms: *N* = 76; Aortic dissections: *N* = 29). P values were determined using one-way analysis of variance (*ANOVA*).

To test whether aortic pathologies lead to specific levels of sVEC, we analyzed the plasma of patients with type B aortic dissection (*n* = 29) and aortic aneurysm (*n* = 76) in comparison to patients with carotid stenosis (*n* = 29) and varicosis (*n* = 24). Western Blot analysis of plasma samples from the different cohorts showed the presence of the cleaved, extracellular domain of VEC (sVEC, 90 kDa) and the absence of the full-length VEC (Fig. 2a). Regarding the absolute sVEC plasma concentrations, we did not find significant differences between the cohorts (Fig. 2b), with an overall sVEC concentration of 3157.5 ± 165.44 ng/mL (mean ± SD). Also, sVEC concentrations, normalized to albumin, showed no significant differences, neither between the two aortic pathologies examined, nor in comparison to varicose patients (Fig. 2d). The significantly lower relative sVEC concentration in carotid stenosis patients is probably attributable to the strikingly ~ 2-fold higher albumin values which we measured in these patients compared to the other disease groups (Fig. 2c). A subgroup analysis of patients with acute aortic dissection (onset of symptoms ≤ 14 days) versus chronic aortic dissection (onset of symptoms ≥ 30 days^17,18^) revealed a trend towards higher sVEC for chronic dissection, though not statistically significantly different (Supplementary Fig. 2a).

## sVEC plasma levels correlate with TNF-α and ADAM10 plasma levels

**Fig. 3:**
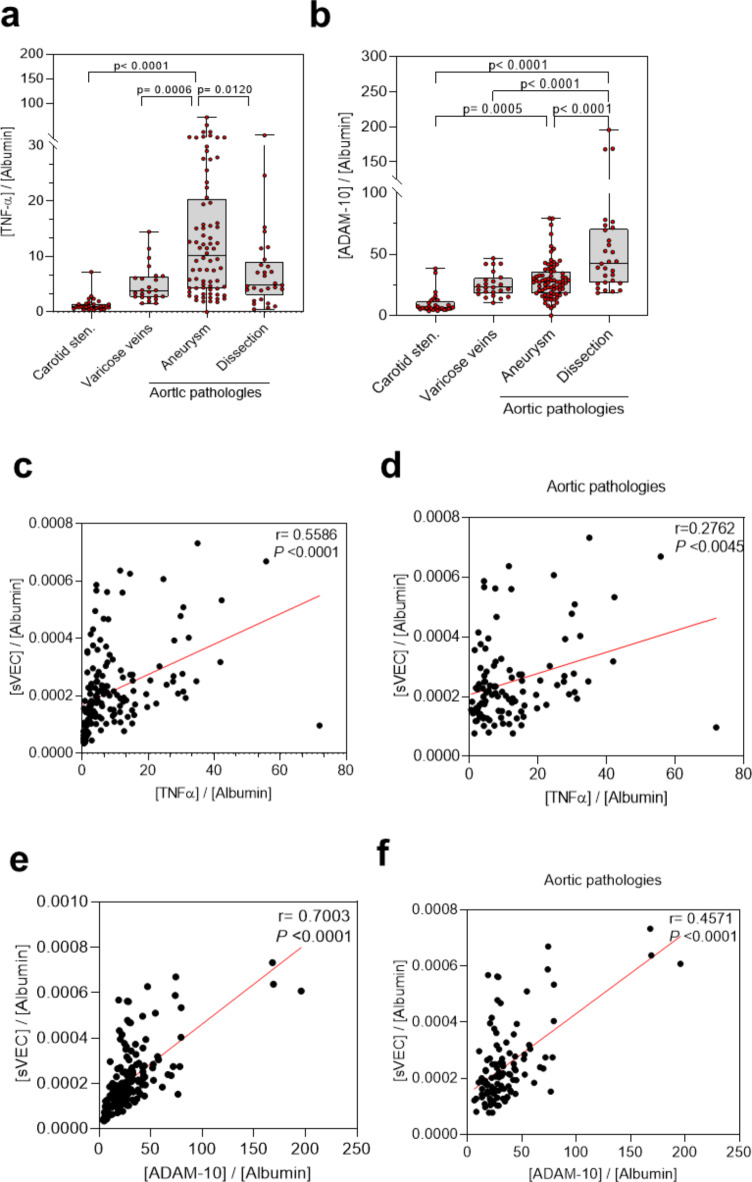
Plasma levels of TNF-α and ADAM10 in patients with different vascular diseases. (**a**,** b**) Albumin-normalized plasma TNF-α (**a**) and ADAM10 (**b**). For normalization albumin concentration as determined in Fig. 2C were used. P values were determined using Kruskal-Wallis test. A total of *N* = 158 plasma samples of individual patients was analyzed (Carotid stenosis: *N* = 29; Varicose veins: *N* = 24; Aortic aneurysms: *N* = 76; Aortic dissections: *N* = 29). (**c-f**) Correlation and simple linear regression (red line) of the influence of TNF-α (**c**,** d**) and ADAM10 (**e**,** f**) on sVEC levels in patients with indicated vascular pathologies. Correlation coefficient is given as Spearman’s r. P values represent a two tailed testing of the null hypothesis that the data were sampled from a population with no correlation between the two variables. *N* = 158 Patients were analyzed of which 105 resembled aortic pathologies.

Since our in vitro data point to a link between TNF-α, ADAM10 and VEC shedding, we quantified the amount of TNF-α and ADAM10 by ELISA in the same set of plasma samples. Of note, preliminary data already revealed that patients with aortic aneurysm peaked out for absolute concentrations of TNF-α at 18.16 ± 16.44 pg/ml (mean ± SD) (Supplementary Fig. 3a), an effect that remains visible for albumin-normalized TNF-α levels (Fig. 3a). Here, the relative mean TNF-α concentration was significantly elevated by 10.8-fold for aortic aneurysms compared to patients with carotid stenosis, varicose veins (2.9-fold) and aortic dissections (1.9-fold), which were just moderately increased (Fig. 3a). A subgroup analysis also showed no substantial differences between chronic versus acute dissection (Supplementary Fig. 2b). The same analysis was performed for circulating ADAM10 and sVEC levels. Here, absolute ADAM10 levels peaked in aortic dissections (Supplementary Fig. 3b), which was also visible for albumin-corrected ADAM10 levels (Fig. 3b). ADAM10 was significantly increased by ~ 3-fold in dissection patients compared to all other disease groups. Among them, patients with aortic aneurysm had lower relative ADAM10 levels than patients with aortic dissection, but still significantly higher levels than patients with carotid stenosis, who did not differ from patients with varicose veins in this regard (Fig. 3b, Supplementary Fig. 3b).

To figure out if the sVEC levels are influenced by TNF-a and ADAM10, as suggested by the in vitro data (Fig. 1), we performed a simple linear regression analysis including all disease categories and separately for the aortic pathologies including aortic dissection and aneurysm (Fig. 3c-f). Thereby we considered the albumin-normalized TNF-α and sVEC concentrations shown in Fig. 3a, b. Looking at the combined correlations analysis for all disease categories, a general, positive influence of TNF-α (*r* = 0.5586, 95%-CI = 0.4369–0.6604; *p* < 0.0001) and ADAM10 (*r* = 0.7003, 95%-CI: 0.6080–0.7739; *p* < 0.0001) on sVEC level was visible (Fig. 3c, e). As sVEC levels for aortic pathologies (and varicose) patients were significantly higher compared to carotid stenosis we additionally performed a regression analysis within this group. Thereby, aortic pathologies show a similar, though less pronounced, positive impact of TNF-α (*r* = 0.2762, 95%-CI: 0.08302–0.4501, *p* = 0.0045) and ADAM10 (*r* = 0.4571, 95%-CI: 0.2856–0.6002; *p* < 0.0001) on sVEC level, than observed in the combined analysis (Fig. 3d, f). As patients with aortic aneurysms and dissections each present with either specifically elevated TNF-α or ADAM10 (Fig. 3a, b), the sVEC level in these patients, though not significantly different, could be influenced to varying degrees by TNF-α and ADAM10. A subgroup analysis within the group of aortic pathologies revealed that ADAM10 showed a higher degree of correlation with sVEC in patients with aortic dissections (*r* = 0.5955, 95%-CI: 0.2826–0.7939, *p* = 0.0007) with highest correlation found in patients with chronic, aortic dissection (*r* = 0.7890, 95%-CI: 0.4304–0.9325, *p* = 0.0013). Accordingly, for aortic aneurysm a lower degree of correlation of ADAM10 with sVEC was found (*r* = 0.4464, 95%-CI: 0.2393–0.6147, *p* < 0.0001). Such a relation was not found for TNF-α. Here, increased TNF-α levels in patients with aortic aneurysm (Fig. 3a) did not translate into a stronger correlation with sVEC levels (*r* = 0.2300, 95%-CI: -0.003590-0.4398, *p* = 0.0471). Moreover, the correlation coefficient was even higher for aortic dissection (*r* = 0.4445, 95%-CI: 0.08190 − 0.07032, *p* = 0.0157) at significantly lower TNF-α levels compared to aortic aneurysms (Fig. 3b).

In summary, our data demonstrate a role for ADAM10 and TNF-α in shedding of VEC in HAOEC. Except for carotid stenosis, no significant differences in sVEC levels were found at baseline, although these appear to be affected to different degrees by TNF-α and ADAM10.

## Discussion

Cleavage of the extracellular domain of VEC is mediated by a variety of cell surface proteases on endothelial cells or neutrophils^19^. Most ectodomain cleaving enzymes belong to the ADAM family and are associated with the onset and progression of aortic aneurysms^5,14^. If shedding of VEC is part of the pathophysiological process or just a passive, bystanding effect is currently unknown. Our data suggest that TNF-α, ADAM10 and ADAM17 are critical factors which impact VEC shedding and decrease of the human, aortic endothelial barrier. Thereby, our in vitro findings extend findings by other groups on venous endothelial cells (HUVEC) which report an elevated EP and transmigration of leucocytes by impaired ADAM10 function and expression^5^. Disturbed endothelial integrity, dysregulation of VEC as well as infiltration of inflammatory cells into the aortic wall promote aortic aneurysm and dissection^20,21^. Of note, sVEC itself disrupts endothelial barrier function^6^. Therefore, VEC shedding appears to play a more active role in aortic pathophysiology than being an incidental effect. The identification of cellular factors that lead to the induction of VEC shedding is important to understand the pathophysiology. Multiple signaling pathways are probably involved in the activation of proteases that cause VEC shedding. Recently it was demonstrated that a complex of coagulation factor XI with plasminogen activator inhibitor 1 and very low-density lipoprotein receptor activates VEGFR2 on the surface of aortic endothelial cells. This triggers a MAPK signaling cascade that promotes active ADAM10 expression^8^. Another study found that TNF-α induced VEC shedding is depending on Src kinase activity, which leads to altered phosphorylation of VEC^22^. We recently demonstrate that the short-chain fatty acid butyrate influences the phospho-status of VEC by regulating Src kinase^23^. To explore how regulation of the cytosolic VEC domain translates into cleavage of the extracellular domain it would be worthwhile to study the impact of butyrate on sVEC production. This is especially indicated since butyrate and butyrate-producing gut microbiota seem to regulate several pathologic processes during formation of aortic aneurysm and dissection, like the infiltration of the aortic wall by neutrophils^24,25^. This finding points to a regulative effect of butyrate on endothelial barrier function and potentially on junctional protein degradation. Trimethylamine-N-oxide (TMAO), another gut microbiota metabolite which is associated with aortic aneurysm formation^26^, was shown to cause endothelial dysfunction and VEC degradation in vitro^27^. It seems plausible that metabolites from the gut microbiota could regulate the extravasation of immune cells into the aortic wall by controlling the shedding of VEC. Given that VEC fragments increase in the supernatant following neutrophil adhesion to TNF-α-stimulated HUVECs^19^, SCFA and TMAO may also target neutrophil (in addition to endothelial) surface-associated protease activity to regulate degradation of VEC and immune cell extravasation. Further studies are required to assess these mechanisms.

Beyond its putative active, involvement in pathophysiological processes, sVEC is discussed as a potential biomarker for a variety of pathological conditions with an inflammatory background involving the vascular system, including coronary atherosclerosis, sepsis, SARS-CoV2 infection/ COVID-19^28^, rheumatoid arthritis, tumor diseases^29^, autoimmune diseases^22,30^ or sepsis^22,31^. Therefore, sVEC appears to be a basal marker rather than a specific marker for inflammatory processes with endothelial involvement. Indeed, in aortic aneurysms and dissections, no specifically elevated sVEC was observed, considering that the significantly low levels found in carotid stenosis are due to the high plasma albumin concentration (Fig. 2c, d). Therefore, the detection of sVEC alone probably has little specificity for aortic pathologies. Neutrophil surface-bound proteases and ADAMs were shown to produce several VEC fragments in vitro^5,19^. In our experiments, we only observed a 90 kDa fragment. However, we cannot exclude the presence of additional VEC fragments in our cell culture supernatants and plasma samples, that may have gone undetected due to the specificity and sensitivity limitations of one-dimensional electrophoresis and antibody-based detection performed in this study. In the future, mass spectrometric analysis could help identify additional fragments or fragment patterns in plasma, potentially aiding in the identification of disease progression or states associated with the expression and activity of relevant proteases. The inclusion of other clinical markers for aortic dissection like D-dimer, C-reactive protein, sELAF, Big ET-1^32, 11^ could further increase the diagnostic value of sVEC for aortic pathologies, as recently demonstrated by the study of Wang et al. for type B aortic dissection^13^. They also pointed out, that elevated serum sVEC and Vinculin levels were significantly associated with a higher risk of visceral malperfusion and refractory pain in TBAD patients, further referring to a clinical relevance in assessing disease severity.

Although research on sVEC clearance is limited, kidney-related diseases may influence sVEC levels. Ebihara et al.^33^ found no differences in plasma sVEC levels before and after renal replacement therapy in sepsis patients. While kidney failure is more common in our patients with aortic diseases, which could be a confounder, patients with varicose veins also had high sVEC levels despite not having kidney-related diseases. Notably, patients with varicose veins in our study cohort are distinguished by a lower age from patients with aortic diseases (49.3 ± 13.97 years). Therefore, an age-specific composition of senescence-related factors in the blood of patients with varicose veins, which differs from that of patients with aortic disease, could create an environment that enhances protease activity or expression. This, in turn, might contribute to the elevated sVEC levels observed in patients with varicose veins. This is supported by a study that demonstrated serum from varicose patients of a similar age to those in our study group (52 ± 15 years) can induce senescence-related dysfunction in the vascular endothelium, leading to both local and systemic pro-inflammatory conditions^34^.

Another influencing factor for sVEC could be the size of vascular lesion that is likely proportional to the degree of endothelial damage, and which is usually larger for aortic pathologies and varicose veins compared to local defects for carotid stenosis. On the other hand, carotid stenosis indicates systemic atherosclerotic processes, suggesting other factors, which contribute to the sVEC levels.

When we compared acute versus chronic aortic type B dissections, chronic dissections showed a tendency towards higher, although not significant sVEC levels. This could potentially be explained by the accumulation of sVEC in patients with chronic dissections over the course of the disease, coupled with a presumed high proteolytic resistance of sVEC. This is supported by the apparent robust levels of sVEC in cell culture supernatants for ≥ 8 h. Also, a status of chronic inflammation could be of relevance when it comes to the triggering of VEC shedding. In this regard, a connection between the disease activity and sVEC levels was shown for rheumatoid arthritis patients and could be conceivable for patients with vascular diseases^22^. While we did not observe relevant differences in TNF-α plasma levels between chronic and acute aortic dissections, inflammation could be focused on intramural areas and therefore not be reliably detectable using plasma measurements.

We found that TNF-α and ADAM10 levels positively correlate with sVEC in the combined analysis including all disease categories, supporting a general mechanistic linkage between sVEC production and TNF-α/ ADAM10, as suggested by our in vitro findings. Although the ADAM10 concentration does not differ significantly between chronic and acute aortic dissection, sVEC appears to be regulated differently. In contrast to acute courses, we found a stronger positive dependence of the sVEC levels on ADAM10 in chronic forms of the disease. However, the isolated influence of TNF-α and ADAM10 on the plasma sVEC especially for aortic pathologies showed just a moderate influence as indicated by an overall low correlation coefficient. Due to the multifactorial character of the pathologies, especially for aortic pathologies, it must be assumed that, yet unknown confounding/ influencing variables have an effect on the sVEC levels. An alternative analysis of the combined influence of both parameters (and other potential factors) on sVEC (e.g., by multiple regression analysis) might reveal a stronger impact on plasma sVEC. However, due to the likely poor predictive power of the regression models (R^2^ = 0.362 and 0.097) a reliable conclusion cannot be drawn from these models, apart from the fact that we did not analyze ADAM10 activity, neither in plasma nor in in the pathologic aortic wall. Another limitation comes with the fact that plasma samples were taken at a single time point before intervention. Given the dynamic nature of (aortic) disease, future studies should include multiple samplings during the course of the disease (also because baseline sVEC values are usually not available in patients) to identify potential disease-specific/ threshold sVEC values. Furthermore, a larger sample size with an adjusted population would benefit the reliability of our findings. Another limitation of our study is the grouping of diseases irrespective of possible variations of the pathophysiological characteristics and clinical presentation^35^.

In our study we present the occurrence of sVEC in plasma of patients with various vascular disease. While the mechanism of VEC shedding in HAOEC seems to be linked to TNF-α and ADAM10, plasma concentrations are likely dependent on a variety of factors and may also differ during the disease process. A systematic clinical trial and functional in vivo studies are needed to investigate the diagnostic value of sVEC and the mechanism of VEC shedding in vascular diseases.

## Materials and methods

### Cell Culture

Primary human aortic endothelial cells (HAOECs) were purchased from PromoCell (Heidelberg, Germany, c-12271). Passages 5 to 7 of HAOECs were cultured at 37 °C and 5% CO2 in a humidified incubator in Endothelial Cell Growth Medium MV (PromoCell, Heidelberg, Germany, C-22020) and Endothelial Cell Supplement Mix (PromoCell, Heidelberg, Germany, c-39225). Cells (1 × 10^5^ cells/well) were seeded in a 24-well-plate (SARSTEDT,83.3924) and grown for ≤ 72 h to 100% confluence. Afterwards, medium was exchanged and HAOECs were either stimulated for the indicated times and concentrations with TNF-α (Thermo fisher scientific, 10756363), or pre-treated for 30 min with ADAM17 Inhibitor (Sigma-Aldrich, USA, 579050), ADAM10 Inhibitor GI254023X (Sigma-Aldrich, USA, SML0789) or vehicle (DMSO, Sigma-Aldrich, USA, D8148). All incubations were carried out at 37 °C and 5% CO2 in a humidified incubator. Supernatants were then collected and kept at -20 °C until analysis.

## Antibodies

We used the following commercially available antibodies: VE-cadherin (E6N7A) (rabbit,1:1000; #93467, Cell Signaling) primary antibody and HRP-linked secondary anti-rabbit antibody (0.5 µL/mL) was used to detect VEC in western blot.

### Western blotting

Western blotting was used to determine protein amount in cell supernatants and patient plasma samples. Cell culture supernatants were collected. The total protein concentration was measured by using the Pierce BCA Protein Assay Kit (Thermo Fisher Scientific, 23225) according to the manufacturer’s instructions. SDS-PAGE sample buffer (250 mM Tris-HCl (pH6.8) (Roth,4855.2), 30%v/v Glycerin (Fisher Scientific, 10021083), 10%w/v SDS, 0.5 M 1,4-Dithiothreitol (DTT) (Roth,6908.1), 0.5%w/v Bromphenol blue (Serva,15375.02)) was added to the supernatants and heated at 100 °C for 10 minutes. Twenty micrograms of total protein from the supernatant was loaded on sodium dodecyl sulfate-polyacrylamide gel electrophoresis (SDS-PAGE) (Resolving gel (2,5 ml running buffer (1.5 M Tris-HCL (pH 9.0) 0.4%v/v TEMED (Roth, 2367.1), 0.4%w/v SDS), 4.02 ml Millipore water, 3.38 ml Rotiphorese ^®^Gel30 (Roth, 3029.2), 45µl 10%v/v Ammonium persulfate (Roth, 9592.5)), stacking gel (4.44 ml stacking buffer (140 mM Tris-HCl (pH 6.8), 0.1%v/v TEMED, 0.1%w/v SDS), 650 µl Rotiphorese ^®^Gel30, 100 µl 10%v/v Ammonium persulfate) and electro-transferred into 0.45 μm Nitrocellulose membrane (Roth, 9201.1) according to the instructions. The membranes were then incubated with 10% low fat milk (MILSANI, Germany) in 1× TBST (1×TBS containing 0.05%v/v Tween-20 (AppliChem, A4974) at room temperature for 1 h. Primary antibody against VEC (clone E6N7A) was diluted in 10%w/v low fat milk in 1×TBST and incubated at 4 °C over night. After washing three times for 10 minutes in 1×TBST, membranes were incubated with a HRP-linked secondary antibody (Cell Signaling, anti-Rabbit IgG HRP-linked antibody, goat, 1:2000, #7074) for 45 minutes at room temperature, followed by detection by enhanced chemiluminescence (Clarity ECL; BioRad, Canada) and imaging on a ChemiDoc Imaging System (BioRad). Protein abundance was quantified by densitometry (Image Lab).

### Enzyme-Linked Immunosorbent Assays (ELISA)

Soluble VE-cadherin was measured in cell supernatants and patients plasma using a commercial ELISA kit (DCADV0; R&D Systems, Wiesbaden-Nordenstadt, Germany). Here a quantitative sandwich enzyme immunoassay technique was applied using a monoclonal antibody specific for human VEC. The assay was performed in a 96-well microplate with removable strips. 100 µl of Assay Diluent was added to each well, followed by 50 µl of standards, controls, or samples. The plate was covered and incubated for 2 h at room temperature. After incubation, wells were aspirated and washed four times with 400 µl of Wash Buffer. Following washing, 200 µL of Human VEC Conjugate was added to each well, and the plate was incubated for another 2 h at room temperature. The aspiration and wash steps were repeated as described previously. Then, 200 µl of Substrate Solution was added to each well and incubated for 30 min at room temperature, protected from light. The reaction was stopped by adding 50 µl of Stop Solution per well. The optical density was measured within 30 min using a microplate reader set to 450 nm, with wavelength correction at 540–570 nm. Albumin, ADAM10 and TNF-α plasma levels were measured using commercial ELISA kits (DY1455, DY936-05, DTA00D; R&D Systems, Wiesbaden-Nordenstadt, Germany). All kits were used according to manufacturer’s instructions as principally described above for VEC. Plasma albumin concentrations were determined, to compensate for different plasma volumes and thus to take into account distorting dilution effects on sVEC, ADAM10 and TNF-α plasma levels. Therefore, we normalized the actual sVEC, ADAM10 and TNF-α concentrations to the measured albumin concentration; similar to Flemming et al.^9^.

### Endothelial permeability assay

HAOECs were seeded on top of a polyethylene terephtalate (PET) membrane transwell insert on 12-well-plates (TC-Inserts, 0.4 μm Pore, Sarstedt, 83.3931.041) at a density of 10^5^ cells/chamber and cultured for 72 h. After discarding the medium, the cells in the upper chamber were incubated with TNF-α (100 ng/ml) for 4 h. Subsequently, the medium in the apical chamber was replaced by endothelial cell basal medium containing 0.05 mg/ml of 10 kDa dextran (Invitrogen, D22910) followed by incubation for 30 min at 37 °C in a CO2 incubator, after which the fluorescence (F) intensity in the upper and lower chambers was determined with a 96-well plate reader (Synergy HTX Multifunction Detector, BioTek, United States: excitation 485/20 and emission 528/20). All independent experiments were performed in triplicate. Permeability was presented as F_basal_/F_apical_ (Fb/Fa).

### Cellular viability assay

Viability of HAOEC upon treatment with various TNF-α concentrations ranging from 100 to 1000 ng/ml was performed by an commercially available “MTT” assay (Promega, G4000, including Solubilization Solution/Stop Mix G401A and Dye Solution G402A) as described by Guo et al.^23^. Briefly, HAOECs (1 × 10^4^ cells/well) were seeded in a 96-well plate and incubated for 48 h. Cells were then stimulated using Basic Endothelial Growth Medium, supplemented or not with the indicated concentrations of TNF-α and further incubated for 4 h. Then, 15 µl of Dye Solution was added to each well, and the plate was incubated for 1 h at 37 °C. Subsequently, 100 µl of Stop Solution was added to each well and incubated for 1 h at room temperature. Wells were mixed thoroughly, and absorbance was measured at 570 nm using a microplate reader to assess cell viability.

### Study design and baseline characteristics of the study cohort

We conducted a case control and outcome oriented observational study with a prospective data collection. The investigation conformed to the principles outlined in the Declaration of Helsinki. An institutional review board approved the study (Ethics Committee of the Westphalia-Lippe Medical Association and the University of Münster, Gartenstraße 210–214, 48147 Münster, Germany, file number 2020-009-f-S), and the participants gave written informed consent. Patients were enrolled from the Clinic for Vascular and Endovascular Surgery of the University Hospital Muenster. Patients selected had to be over the age of 18 years without any connective tissue disorder. Pregnant patients were excluded from our study. Patients were divided in two control groups (varicosis and carotid stenosis) and a group of aortic pathologies (including aortic aneurysm and aortic dissections) with characteristics as described below (Table 1).

**Table 1:** Characteristics of patients included in the study. P-value represents Chi-square test and Kruskal-Wallis test (age). Statistically significant p-values are marked in bold. ACEIs = angiotensin-converting enzyme inhibitors. COPD = chronic obstructive pulmonary disease. AT-1-inhibitors = angiotensin-II-receptor-type-1-antagonists. ASA = acetylsalicylic acid.

Vascular disease	total	Dissection	Aneurysm	Carotid sten.	varicose veins	*p*-value
Patients, n(%)	158	29	76	29	24	
Gender						**0**,**007**
Women	51 (32,2)	8 (27,5)	20 (26,3)	8 (27,6)	15 (62,5)	
Men	107 (67,7)	21 (72,4)	56 (73,7)	21 (72,6)	9 (37,5)	
Age, mean (standard deviation)	66,12 (12,77)	65 (11,37)	70,5 (9,44)	69,5 (8,96)	49,3 (13,97)	**0**,**001**
Autoimmune disease, n(%)	7 (4,4)	0 (0)	6 (7,9)	0 (0)	1 (4,2)	0,183
Atrial fibrillation, n(%)	26 (16,4)	5 (17,2)	16 (21,1)	4 (13,8)	1 (4,2)	0,265
Heart failure, n(%)	5 (3,1)	0 (0)	3 (3,9)	2 (6,9)	0 (0)	0,361
Diabetes mellitus type 2, n(%)	20 (12,6)	3 (10,3)	8 (10,5)	7 (24,1)	3 (12,5)	0,296
Hypertension	106 (66,4)	24 (82,7)	57 (75)	22 (75,9)	4 (16,7)	**0**,**001**
COPD	6 (3,7)	1 (3,4)	5 (6,6)	0 (0)	0 (0)	0,294
Medication						
Statines	85 (53,7)	11 (37,9)	45 (59,2)	28 (96,5)	3 (12,5)	**0**,**001**
ACEIs	86 (54,4)	10 (34,4)	45 (59,2)	28 (96,6)	3 (12,5)	**0**,**001**
Betablockers	47 (29,7)	10 (34,4)	26 (34,2)	11 (37,9)	1 (4,2)	**0**,**025**
AT-1-inhibitors	85 (53,7)	21 (72,4)	41 (53,9)	21 (72,4)	2 (8,3)	**0**,**001**
ASA	41 (25,9)	8 (27,5)	22 (28,9)	10 (34,5)	1 (4,2)	0,059
Anticoagulants	104 (65,8)	22 (75,8)	61 (80,3)	21 (72,4)	1 (4,2)	**0**,**001**
Coronary heart disease	35 (22,1)	5 (17,2)	19 (25)	11 (37,9)	0 (0)	**0**,**008**
Stroke	17 (10,7)	1 (3,4)	9 (11,8)	6 (20,7)	1 (4,2)	0,123
Peripheral artery disease	17 (10,7)	1 (3,4)	12 (15,8)	4 (13,8)	0 (0)	0,078
Renal insufficiency, n(%)	23 (14,5)	5 (17,2)	16 (21,1)	2 (6,9)	0 (0)	**0**,**042**

### Plasma collection and preparation

To obtain plasma samples, the EDTA whole blood was centrifuged at 1000g and 4 °C, for 10 min. The supernatant (plasma) was transferred into a 15 ml tube then swirled and aliquoted into 1.8 ml cryotubes (Thermo Scientific) and stored at -80 °C.

### Statistical analysis

Statistical analysis was conducted using GraphPad Prism 9 software (GraphPad Software, San Diego, CA, United States) and SPSS Statistics for Windows version 26.0 (IBM Corp., Armonk, NY, USA). Shapiro–Wilk test was used to assess normal distribution and equal variance. To compare multiple experimental groups, parametric data were analyzed with one-way analysis of variance (*ANOVA*) and non-parametric data with Kruskal-Wallis test, while an unpaired t-Test (*Welch’s t-test*) was used to compare two independent samples. Simple linear regression and correlation analysis were performed with GraphPad Prism 9 software (GraphPad Software, San Diego, CA, United States). Non-parametric Spearman correlation (r) was computed, to map the degree of correlation between plasma ADAM10, TNF-α and sVEC. Characteristics of patients (Table 1) were analyzed by Chi-square and Kruskal-wallis test (age) using SPSS software (IBM). Statistical significance was considered for *p* ≤ 0.05.

## Electronic Supplementary Material

Below is the link to the electronic supplementary material.

Supplementary Material 1

## Data Availability

The datasets generated and/or analyzed in this study are available from the corresponding author on reasonable request.
