# Supplementary material for: VE-cadherin shedding in vitro and in patients with aortic aneurysm and dissection
Source: Sci Rep. 2024 Nov 5;14:26743. doi: 10.1038/s41598-024-77940-3 (PMC11538497; doi:10.1038/s41598-024-77940-3)
Supplement: Supplementary file 1 — Supplementary Material 1 [file 41598_2024_77940_MOESM1_ESM.docx]

##### Supplementary Information

##### VE-Cadherin shedding in vitro and in patients with aortic aneurysm and dissection

Paul Stammer, Inka Terhorst, Dr. med. Jiangang Guo^#^, Dr. med. Abdulhakim Ibrahim, Univ.-Prof. Dr. med. Alexander Oberhuber, Dr. rer. nat. Thorsten Eierhoff*

Clinic for Vascular and Endovascular Surgery, University Hospital Münster, Albert- Schweitzer-Campus 1, 48149 Münster

^#^current address: Department of Endovascular and Vascular Surgery, Affiliated Hospital of Guilin Medical University, Guilin, Guangxi, China.


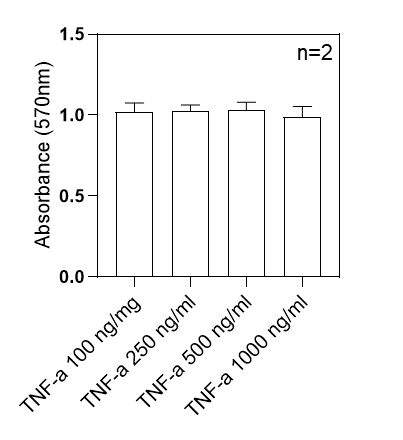


**Supplementary Figure 1**: **Cytotoxicity of various concentrations of TNF-α.** TNF-α was incubated for 2h with HAOEC and subsequently analyzed by the MTT assay according to the manufacturer’s instructions. Absorbance values were normalized to untreated control group. N = 2 independent experiments with each 5 biological replicates were conducted. P values were determined using one-way analysis of variance (*ANOVA*) indicating no significant differences between groups.


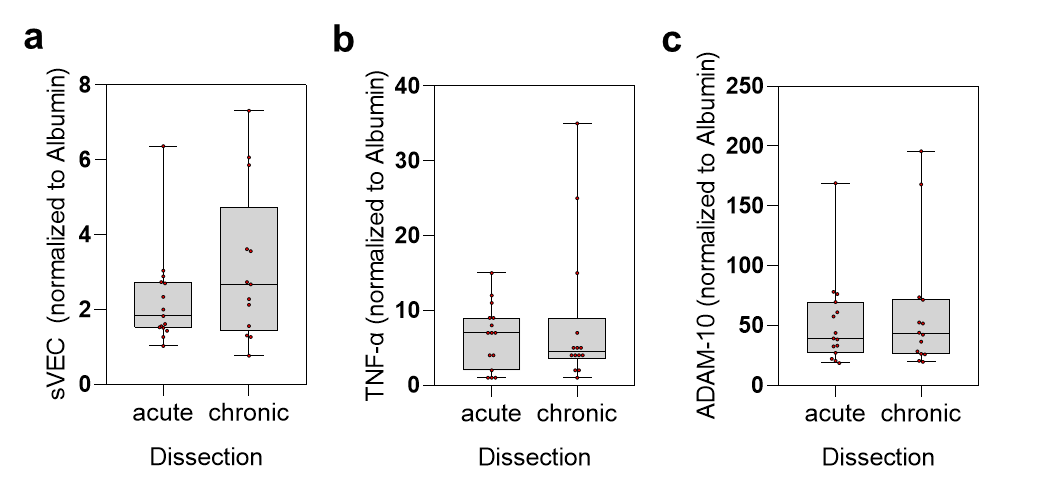


**Supplementary Figure 2: sVEC, TNF-α and ADAM10 plasma level in acute versus chronic Stanford type B aortic dissection.** Albumin-normalized TNF-α, albumin and sVEC level extracted from data shown in Fig. 2b and 3a for acute (N=15) and chronic (N=14) pathologies are shown. P values were determined using an unpaired t-Test (*Welch’s t-test*) indicating no significant differences between groups.


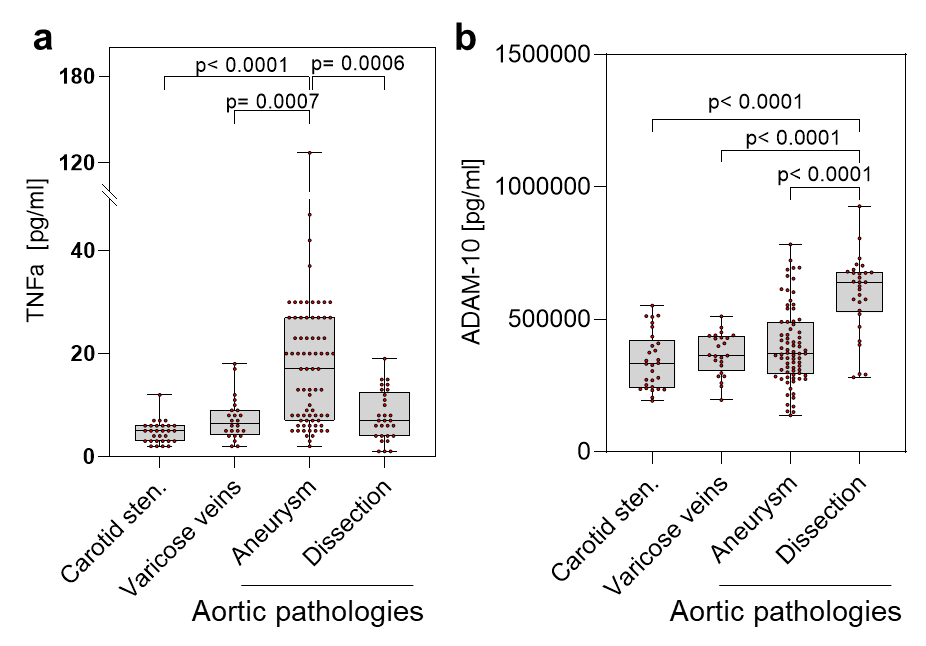


A

B

Aortic pathologies

Aortic pathologies

A

B

Aortic pathologies

Aortic pathologies

**Supplementary Figure 3: Absolute concentrations of plasma TNF-α (a) and ADAM10 (b) determined by ELISA.** A total N = 158 plasma samples of individual patients was analyzed (Carotid stenosis: N=29; Varicose veins: N=24; Aortic aneurysms: N=76; Aortic dissections: N=29). Kruskal-Wallis test was used to determine statistical significance. Statistical significance was considered for p ≤ 0.05 (*), p ≤ 0.005 (**), p ≤ 0.001 (***) and p ≤ 0.0001 (****).

**a b**


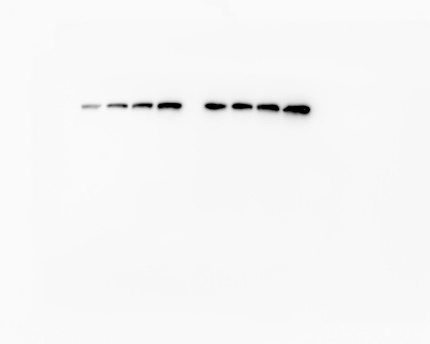

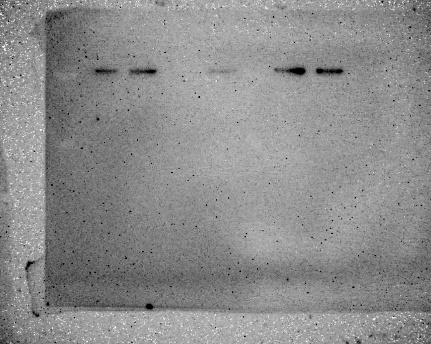


**Supplementary Figure 4: Uncropped blots corresponding to Figure 1d (a) and Figure 2a (b).** Areas outlined in red are shown in the corresponding main figure.
